# Developmental toxicity and physicochemical characterization of cisplatin–loaded ginger oil nanoemulsion in casper zebrafish

**DOI:** 10.3389/ftox.2026.1862521

**Published:** 2026-07-01

**Authors:** A. N. K. V. Sravani, John Thomas

**Affiliations:** Center for Nanobiotechnology, Vellore Institute of Technology, VIT University, Vellore, India

**Keywords:** casper zebrafish, developmental toxicity, embryo toxicity, embryogenesis, nanoemulsion toxicology

## Abstract

**Introduction:**

This study used Casper zebrafish embryos and larvae as a model to examine the creation and safety evaluation of a cisplatin-loaded ginger essential oil nanoemulsion (Cisp-GE-NE).

**Methods:**

The Cisp-GE-NE was made using an oil-in-water ultrasonic dispersion technique with Tween 80, and it was characterised using FTIR analysis, DLS, zeta potential, and UV-visible spectroscopy. A sustained release of cisplatin was noted at pH values of 3.5, 5.5, and 7.4.

**Results:**

With LC50 values of 5.3 μg/mL, 14.4 μg/mL, and 3.7 μg/mL, zebrafish embryos exposed to different concentrations of Cisp-GE-NE showed decreased epiboly progression, morphological abnormalities, and significant mortality. Spinal curvature, pericardial oedema, smaller eyes, reduced swim bladder inflation, and decreased body length were among the most notable larval defects. Neurodevelopmental toxicity was indicated by behavioural studies that revealed different swimming patterns in light and dark environments.

**Discussion and Conclusion:**

The results draw attention to the possible toxicity of nanoemulsion systems based on ginger oil and stress the significance of conducting a safety assessment before their use in clinical or environmental settings.

## 1 Introduction

Essential oils are commonly used in the food, cosmetic, and pharmaceutical industries for their biological properties, including antimicrobial and antioxidant activity (Bassolé and Juliani, 2012). Nevertheless, the applications of essential oils are hindered by several challenges, including low solubility, volatility, and bioavailability. Hence, several researchers suggest that the integration of these essential oils as nanoemulsions presents a compelling solution to the challenges faced by traditional applications of essential oils. By enhancing solubility, bioavailability, and stability, nanoemulsions can unlock the full potential of essential oils across a range of industries. Nanoemulsions are clear or translucent emulsions with droplet sizes generally ranging from 20 to 500 nanometers. Due to their tiny droplet size, nanoemulsions have unique properties that enhance their stability, bioavailability, and effectiveness in drug delivery applications, cosmetics, and food products. However, the extensive application of nanoemulsions prepared from essential oils, in phytotherapy, as well as other applications, may also have negative health benefits if not used appropriately. The real fact is that any medicinal herb, depending on dosage and duration of exposure, may have toxic effects at higher concentrations (Bhavaniramya et al., 2019; Kaur et al., 2024). However, the potentially toxic effects of these essential oil-derived nanoemulsions on living organisms have been poorly investigated. Numerous plant-derived essential oils and their nanoformulations have the potential to cause mutagenic, teratogenic, and carcinogenic effects in humans (Jin et al., 2016; da Silva et al., 2023). Therefore, it is essential to conduct toxicity testing in a variety of *in vitro* investigations utilizing animal models, which include experimental screening techniques to ascertain their safety profile. For example, a study evaluating the LD_50_ on treatment in numerous rodents was conducted to determine the acute oral toxicity of various medications and herbs. Despite this, toxicity in embryonic development has historically been studied in rodents, rabbits, and sheep. However, it takes time and money to study these animals. To investigate the toxicity of essential oil-derived nanoemulsions in embryonic development, an alternative animal model is required.

In addition to saving money and time, alternative vertebrate models like zebrafish (*Danio rerio*) are being employed more frequently to adhere to the 3 R principle (Replacement, Reduction, and Refinement), which highlights ethical responsibility in experimental research. The zebrafish (*Danio rerio*) is a well-known vertebrate model. With over 70% similarity to the human genome, the fully sequenced zebrafish genome is an effective tool for researching human disease causes. (Lachowicz et al., 2021; Lammer et al., 2009; Mendis et al., 2018; Karnchanatat et al., 2011; Dasari and Bernard Tchounwou, 2014; Siddik, 2003). It is also being used in pre-clinical studies and toxicology due to its favorable traits (Elmorsy et al., 2024; Alharbi et al., 2023). The unique feature of Zebrafish is having low housing costs and a small size, which makes them an ideal animal model for testing (Lachowicz et al., 2021; Siddik, 2003). They have a high fecundity rate, where a female produces around 300 eggs. (Lachowicz et al., 2021; Shahrajabian et al., 2019). Due to their external development and robustness, zebrafish eggs are well-suited for high-throughput applications and are simple to handle. Zebrafish embryos also have the benefit of being optically transparent, which makes it possible to see development and organogenesis in real time without removing the chorion. The developing zebrafish’s optical transparency enables detailed visual analysis, including fluorescent and Other markers (Lammer et al., 2009; Siddik, 2003). Zebrafish can also develop quickly, with the basic body plan established by 24 h post-fertilization (hpf), embryogenesis completed by 72 hpf, and most organs fully developed by 96 hpf. They reach adulthood in approximately 3 months (Lammer et al., 2009). This makes them suitable for a wide range of toxicological applications throughout their lifespan.

Cisplatin (cis-diamminedichloroplatinum (II)) is a commonly used chemotherapeutic drug that is highly effective in treating a variety of solid tumors, such as non-small cell lung cancers, testicular, ovarian, bladder, and head and neck cancers. Its primary mechanism of cytotoxicity is DNA crosslinking, which impairs transcription and DNA replication and causes rapidly dividing tumor cells to undergo apoptosis. However, several issues, most notably its dose-limiting toxicities, severely limit the practical usefulness of cisplatin. nephrotoxicity, neurotoxicity, hepatotoxicity, ototoxicity, and gastrointestinal disturbances are common side effects that restrict its long-term usage and call for cautious dosage and clinical monitoring. Furthermore, its therapeutic efficacy is further diminished by inherent and acquired resistance mechanisms, such as improved DNA repair, greater drug efflux, and decreased drug accumulation. Drug delivery methods based on nanotechnology, such as cisplatin-loaded nanoemulsions, have been investigated to improve tumor selectivity, decrease systemic toxicity, and increase bioavailability to overcome these restrictions. With droplet sizes in the nanoscale range, nanoemulsions are thermodynamically stable colloidal systems that provide targeted delivery, controlled release, and enhanced solubility of hydrophobic medications like cisplatin.

Ginger, derived from Zingiber officinale Roscoe, is a popular spice and traditional remedy for pain, inflammation, and digestive issues (Jayasinghe and Jayawardena, 2019). Ginger oil is extracted from the fresh rhizomes of Zingiber officinale (Lammer et al., 2009; Mendis et al., 2018). The aroma and flavor are similar to those of the spice, but less potent (Karnchanatat et al., 2011). Ginger essential oil has also been discovered to have antibacterial, antiviral, and Antifungal properties (Dasari and Bernard Tchounwou, 2014). Ginger oil contains monoterpenes like phellandrene, camphene, cineole, linalool, limonene, citral, geraniol, citronellol, and borneol, as well as sesquiterpenes like zingiberene, arcurcumene, bisabolene, sesquiphellandrene, zingiber, and zingiberenol (Siddik, 2003). It contains anticancer compounds, including terpenoids, phenylpropanoids, flavonoids, and sesquiterpenes (Elmorsy et al., 2024). Bioactive chemicals in this oil, including 6-gingerol and zerumbone, have been shown to induce apoptosis in cancer cells (Alharbi et al., 2023). In recent years, researchers have turned to nanoemulsion technology to enhance the therapeutic potential of ginger oil for clinical applications. Nanoemulsions of ginger oil offer advantages such as Enhanced solubility and stability of hydrophobic compounds, increased permeability across biological membranes, Controlled and sustained drug release, and reduced toxicity and side effects. Ginger oil nanoemulsions have shown promise in treating inflammatory diseases such as arthritis, due to their ability to inhibit key inflammatory pathways like COX-2 and TNF-α (Shahrajabian et al., 2019). Nano-formulation ensures better absorption and longer-lasting effects compared to traditional forms. Nanoemulsified ginger oil can be used for conditions like nausea, vomiting, and irritable bowel syndrome (IBS), offering rapid relief and reduced gastric irritation. Ginger oil nanoemulsions can be engineered to deliver anticancer agents more precisely to tumor sites (Munda et al., 2018; Farouk et al., 2022). Their small particle size allows for better tumor penetration, reduced clearance by the immune system, and minimized off-target effects. When combined with conventional chemotherapy drugs, ginger oil nanoemulsions can potentially enhance drug efficacy while lowering required dosages, thus reducing chemotherapy-induced toxicity (Ginger Processing in India Zingiber officinale: A Review, 2018).

Despite the increased interest in nanoemulsion-based drug delivery methods, very few studies have looked into their possible developmental toxicity, especially when chemotherapeutic drugs like cisplatin are coupled with plant-derived essential oils. Furthermore, there have been no *in vivo* animal model studies to evaluate the physicochemical features and toxicological profile of cisplatin-loaded ginger essential oil nanoemulsions, including their embryotoxic and teratogenic effects. The current study aims to evaluate the physicochemical features of cisplatin-loaded ginger essential oil nanoemulsion (Cisp-GE-NE), and cisplatin including particle size, surface charge, and pH-dependent drug release, as well as assess its developmental toxicity using the Casper zebrafish model. The study’s findings provide crucial baseline data for assessing the safety of ginger oil-based nanoemulsion drug delivery systems in future biological applications.

## 2 Materials and methods

Ginger essential oil (99.0% purity) and cisplatin (90.0% pure) were purchased from Sigma Aldrich. Tween 80 (99.0% purity) was purchased from Hi-media. All other reagents utilized were of analytical grade. Stock solutions of Ginger essential oil nanoemulsion-loaded cisplatin were made fresh before the experiments and dissolved in 0.1% (v/v) in the embryonic medium.

### 2.1 Preparation of GE-NE using tween 80 surfactant

The ginger essential oil nanoemulsion (NEs) was prepared by the phase inversion temperature method (PIT) according to the protocol adopted by M. Firoozi et al., with slight modifications (Firoozi et al., 2020). Briefly, the ginger oil nanoemulsion (GE-NE) formulation was carried out by using a magnetic stirrer Spin 4,010 (Tarsons Products Pvt. Ltd., India). Water was added dropwise at room temperature while being continuously stirred at 300 rpm to create the ginger oil nanoemulsion (GE-NE). The prepared GE-NEs had an oil-to-water ratio (organic phase to aqueous phase) that ranged from 1:1 to 1:4. For 4 weeks, the prepared GE-NE were kept at various temperatures: room temperature (dark and light), and 37 °C (dark and light). The GE-NE that demonstrated long-term kinetic stability are selected for further characterization and experiments (Anarjan et al., 2014).

### 2.2 Preparation of ginger oil nanoemulsion loaded with cisplatin

Cisplatin was loaded into the core of GE-NE by dispersing Cisplatin, i.e., 1 mg w/v in 20 mL of ginger oil nanoemulsion prepared by ultrasonication and further made up with water (v/v), reaching a final volume of 100 mL. The complete setup is later placed in an ultra-shaker water bath, maintaining a temperature range of 37 °C for 30 min. The procedure was repeated for other cisplatin concentrations (100, 150, 200, and 250 μg/mL). Later, the uniquely dispersed solutions were ultrasonicated for 30 min in a 750-W ultrasonicator, maintaining 15 s on pulse and 15 s off pulse with 40% amplitude (Mohamed and El-Naggar, 2018). The undispersed drug was later removed by filtering with a 0.45 μm polyvinylidene difluoride (PVDF) membrane. Further concentration of the drug in the oil-water phase was measured using UV-visible spectroscopy at 270 nm.

### 2.3 Physical-chemical characterization of ginger oil nanoemulsion loaded with cisplatin

#### 2.3.1 DLS

The mean particle size including the emulsifier layer (Horiba Scientific- SZ-100), zeta potential value, polydispersity index (PDI), and particle size distribution (PSD) of GE-NE were assessed using a dynamic light scattering practical analyzer (DLS) at 37 °C. The stability of the synthesized GE-NE was assessed by monitoring the change in nanoemulsion droplet size over 4 weeks during storage at room temperature (light and dark) and 37 °C (light and dark) temperature (Mohamed and El-Naggar, 2018).

#### 2.3.2 Fourier-transform infrared spectroscopy (FTIR)

FTIR (Jasco) analysis was performed according to protocol by Farshbaf-Sadigh et al. (2021) to determine the successful functionalization of GE-NE, Cisp, and Cisp-GE-NE at each phase. FTIR of Cisplatin, and Cisp-GE-NE were compared before and after covalent modification for each compound spectra with a resolution of 4 cm^-1^ spanning the wavenumber range of 400–3,000 cm^-1^ using the KBr pellet disk method (Farshbaf-Sadigh et al., 2021).

#### 2.3.3 Stability and drug release kinetics of OH-SWCNT-cisp-GE-NE

*In vitro* drug release investigations were conducted using dialysis tubing cellulose membranes from (Sigma Aldrich-Merck, Country) that had a capacity of 10 mL (w/v) with an average flat diameter of 10 mm. briefly The dialysis tube was filled with Cisp-GE-NE, and the tubing was immersed in 50 mL of 1x stimulated gastrointestinal fluid at three different pH 3.4, 6.5 and 7.4 to perform dialysis separately to maintain the ambient temperature, i.e., 37 °C and to maintain a constant volume. The solution was constantly stirred with a magnetic stirrer and covered with a thin layer of Parafilm to reduce evaporation. 3 mL of release medium is collected by replacing it with 3 mL of the gastrointestinal fluid at predetermined intervals, as follows (Sravani et al., 2023). The amount of drug released in each sample was determined using UV-visible spectroscopy at between 250 nm and 270 nm. The following Equation 1 was used to express cumulative drug release versus time.

$$\text{Cumulative Drug Release}\left(\%\right)=\left(\text{Amount of drug released}/\text{Total amount of drug loaded}\right)\times \;100$$ (1)

The stability of Cisp-GE-NE was carried out for 4 weeks, by placing all concentrations of the Cisp-GE-NE sample at room temperature and 37 °C in both light and dark conditions. After 4 weeks, the percentage of Cisp-GE-NE was calculated and contrasted with the initial total concentrations.

### 2.4 Zebrafish husbandry and embryo collection

All experiments were performed in the wet lab conditions. Adult zebrafish AB strains were raised and maintained in an automated recirculating system under standard conditions for Casper-strained zebrafish (system water at 28 °C ± 2 °C, pH = 6.5–8.5, and conductivity between 600 and 700 μS) with a 14/10 light/dark photoperiod. Every experimental procedure complied with OECD guideline 236 (Fish Embryo Acute Toxicity Test) (da Silva et al., 2023; Oliveira et al., 2020; OECD, 2013). Embryos were obtained by crossing adult females and males at a 2:1 male to female ratio in a breeding tank. Fertilized embryos were collected after natural laying and subsequently screened under a stereomicroscope for further experiments (Oliveira et al., 2020). Fertilized eggs were checked and collected 20 min after crossing and incubated in embryo medium. Only embryos that had normal development were used in the experimental groups. At 2 hpf (hours postfertilization), embryos were transferred to Petri dishes for further experiments. And analyzed for their teratogenic activity, like mortality, morphometry, and embryotoxicity.

### 2.5 Exposure of cisp-ge-ne nanoemulsion system

To ensure compatibility with zebrafish developmental requirements, the Cisp-GE-NE was initially suspended in embryo medium (RO-water). Embryos of the Casper zebrafish strain were exposed to varying concentrations (100, 150, 200, and 250 μg/mL) of Cisp-GE-NE. The test formulations were applied to embryos at 1 hpf (hour post fertilization), and they were monitored until 120 hpf. The exposure media were renewed daily to maintain stability and consistency. The embryos were housed in 24-well culture plates, with four embryos per well, and maintained in an incubator under controlled conditions of temperature, humidity, and photoperiod. Each treatment group consisted of a minimum of 32 embryos and was conducted in duplicate to ensure reproducibility. For comparative toxicity assessment, additional embryo groups were exposed to free cisplatin under identical experimental conditions for LC50 determination.

### 2.6 Measurement of epiboly

Epiboly, characterized by the movement and expansion of cells over the yolk cell, was assessed at 8 hpf, corresponding to 6 h of treatment exposure. A total of 60 embryos (n = 10 per group across 6 experimental groups) were fixed overnight in 4% paraformaldehyde (PFA) prepared in phosphate-buffered saline (PBS). Each embryo was considered a biological replicate, and the experiment was independently repeated two times to ensure reproducibility. Fixed embryos were imaged using a stereomicroscope (Leica DFC 450C), and the degree of epiboly progression was quantified. Measurements were performed using ImageJ software (version 1.53 k, 2019, National Institutes of Health, Bethesda, MD, United States), which was used to calculate epiboly percentage by measuring the extent of yolk coverage by using the formulea described by Anand David et al. (2016).

Distance of blastoderm margin from vegetal pole÷yolk diameter×100

### 2.7 Embryonic development/teratogenicity

The developmental parameters of Casper zebrafish embryos were monitored daily, with specific attention to mortality, hatching rates, and morphological deformities along the body axis. Observations were conducted using a stereomicroscope (Leica DFC 450C). Embryonic and larval mortality was assessed each morning, and any non-viable specimens were promptly removed to prevent interference with the remaining population.

#### 2.7.1 Evaluation of mortality

The potential mortality associated with Cisp-GE-NE was assessed in Casper zebrafish embryos, with 20 embryos allocated to each of six experimental groups, totalling 120 specimens. Embryos were examined daily under a stereomicroscope (Leica DFC 450C) up to 6 dpf (days post fertilization). In order to observe delayed morphological recovery beyond the OECD 236 standard (96 hpf), embryos were observed up to 120 hpf or 5 dpf. Post-hatching development was evaluated to ensure humane endpoints, regulatory compliance, and data reliability (Anand David et al., 2016) to ensure regulatory compliance and data reliability. The survival rate in the control group remained above 90% throughout the experimental period, thereby validating the experimental conditions and findings. Teratogenic effects were evaluated qualitatively through high-resolution imaging, as described in the section. Observed malformations included ocular anomalies, pericardial and generalized edema, axial skeletal deformities (including tail and spinal curvature), and locomotor system impairments (da Silva et al., 2023; Anand David et al., 2016; Cadena et al., 2020; Cadena et al., 2022).

#### 2.7.2 Morphometry

Hatching marks the critical transition from the embryonic stage to a free-swimming larval stage and serves as an indicator of developmental progression over time. Hatching rates were monitored daily until complete hatching was observed in all viable specimens. Morphometric analysis was conducted using zebrafish larvae at 6 dpf, with 20 larvae per group across six experimental groups (n = 120). Larvae were fixed in 4% paraformaldehyde (PFA) before imaging. Due to elevated mortality at higher treatment concentrations, only groups exposed to the lowest concentrations of each essential oil were included in the morphometric evaluation. Lateral-view images were acquired using a stereomicroscope, as previously described. Morphometric parameters, including eye length (EL), head length (HL), head width (HW), and standard body length (SL), were measured using ImageJ software (version 1.53 k), following the methodology outlined by Cadena et al. (2022).

#### 2.7.3 Embryo toxicity

The median lethal concentration (LC_50_) was calculated using probit analysis, following the methodology outlined by da Silva et al. (2023). LC_50_ values were estimated based on the survival data of zebrafish larvae at 6 dpf, after exposure to different concentrations of cisp, GE-NE, and the Cisp-GE-NE nanoemulsion formulations.

### 2.8 Statistical analysis

One-way analysis of variance (ANOVA) and Tukey’s post hoc test were used for statistical comparisons between several groups. Kaplan-Meier curves were used for survival analysis, and the log-rank test was used for comparison. Probit analysis was used to produce. LC_50_ values with 95% confidence intervals. Statistical significance was defined as a p-value of less than 0.05. All data are presented as mean ± standard deviation (SD).

## 3 Results

### 3.1 Ginger oil nanoemulsion preparation

**FIGURE 1:**
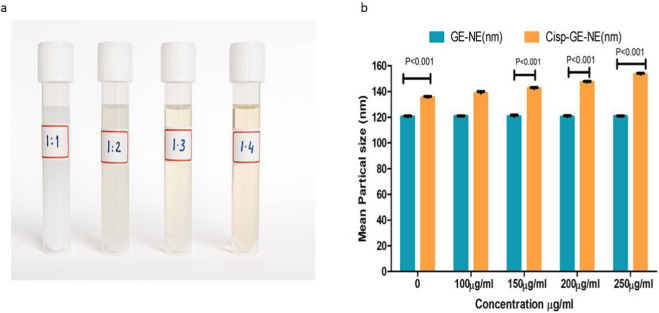
**(a)** Visual appearance of NE formulations prepared at varying oil-to-surfactant ratios (1:1, 1:2, 1:3, and 1:4). Increasing surfactant concentration resulted in improved clarity, indicating enhanced emulsification and reduced droplet size. The 1:4 ratio yielded the most transparent and stable nanoemulsion, suggesting an optimal formulation for drug delivery applications. **(b)** Mean particle size analysis of blank GE-NE and Cisp-GE-NE at increasing cisplatin concentrations (0–250 μg/mL). A concentration-dependent increase in particle size was observed upon drug loading, with all Cisp-GE-NE samples showing significantly larger sizes compared to GE-NE (P < 0.001). This indicates successful cisplatin incorporation without compromising nanoemulsion stability, maintaining sizes within the nanometric range favorable for drug delivery. Data are presented as mean particle size (nm) ± SD. A statistically significant increase in particle size is observed in Cisp-GE-NE compared to blank GE-NE across all concentrations (P < 0.001).

The ginger essential oil nanoemulsions (GE-NE) prepared via the PIT method exhibited notable physicochemical stability under various storage conditions. Among the tested formulations, those with oil-water ratios of 1:3 and 1:4 demonstrated the most stable characteristics and features when compared to 1:1 and 1:2, with no visible phase separation or creaming during the 4-week observation period, as shown in Figure 1a.

Kinetic stability was assessed for all four formulations, and results showed that the GE-NE formulations (1.1,1.2,1.3 and 1.4), which are stored at room temperature in the dark, maintained optimal clarity and homogeneity when compared to those exposed to light (both at room temperature and 37 °C), displayed minor turbidity after 3 weeks, indicating the onset of destabilization. Samples stored at 37 °C in the dark showed slightly improved thermal resilience compared to those exposed to light at the same temperature, although a minimal reduction in clarity was observed by the end of the fourth week. The Kinetic stability of all formulations is represented in Table 1. Overall, GE-NE stored at room temperature in the dark exhibited the highest kinetic stability, making them suitable candidates for further physicochemical characterization.

**TABLE 1:** The stability profile of the nanoformulation was stored under four different conditions over 4 weeks: Room Temperature (Dark), Room Temperature (Light), 37 °C (Dark), and 37 °C (Light). Data represent the mean relative concentration of the active compound (n = 3) normalized to the initial value (Week 1 = 1.00). A significant decrease in stability was observed in samples exposed to light, especially at elevated temperatures (37 °C), with only 65% of the active compound remaining by Week 4. One-way ANOVA followed by Tukey’s post-hoc test revealed a statistically significant reduction in stability under light-exposed conditions at both room temperature and 37 °C compared to dark storage conditions (P < 0.01). No significant difference was observed between Room Temp (Dark) and 37 °C (Dark) conditions over the 4 weeks (P > 0.05), indicating that temperature alone had a minor effect compared to light exposure.

Week	Room temp (dark)	Room temp (light)	37 °C (dark)	37 °C (light)
1	1.00	0.95	0.97	0.92
2	0.99	0.90	0.95	0.85
3	0.98	0.80	0.93	0.75
4	0.97	0.70	0.90	0.65

### 3.2 Formulation and encapsulation efficiency of Cisp-GE-NE

Cisplatin was successfully loaded into GE-NE using an oil-in-water dispersion method followed by ultrasonication. The formulations were prepared at various cisplatin concentrations (100, 150, 200, and 250 μg/mL). Post-ultrasonication, the solutions appeared visually homogeneous, with no visible phase separation, indicating successful emulsification. The undispersed drug was filtered and further quantified with UV-Visible spectroscopy to identify the concentration of cisplatin in the oil-water phase. The maximum absorption peak for cisplatin was observed at 270 nm. Drug encapsulation is the crucial factor for developing an effective nanocarrier system (Mura et al., 2013). The oil-water nanoemulsion system is said to carry 95% of hydrophobic drugs into its core for targeted drug delivery. Hence, this made us choose the ginger oil-water emulsion system for the current study. From our results, the encapsulation efficiency of cisplatin loaded into the core of ginger oil-water nanoemulsion was observed for different concentrations like 100 μg/mL, 150 μg/mL, 200 μg/mL, and 250 μg/mL which showed the highest encapsulation efficiency (EE%) at 150 μg/mL, i.e., (81.5% ± 1.8%) beyond which a slight reduction in EE% was observed as shown in Table 2. This may be attributed to the saturation of the oil core or the limited solubility of cisplatin within the nanoemulsion system (Qiang et al., 2020). The encapsulation efficiencies for the respective formulations are shown in Table 2.

**TABLE 2:** Encapsulation efficiency (%) of cisplatin-loaded nanoemulsions at various drug concentrations (100–250 μg/mL). Data are expressed as mean ± standard deviation (SD), based on triplicate measurements (n = 3). The encapsulation efficiency showed a concentration-dependent trend, with the highest efficiency observed at 150 μg/mL (81.5% ± 1.8%), suggesting optimal drug entrapment at this loading. A decline in encapsulation was noted at 250 μg/mL (73.4% ± 2.9%), indicating possible saturation of the nanoemulsion system or drug precipitation at higher concentrations. Statistical analysis using one-way ANOVA revealed significant differences between groups (P < 0.05), particularly between 150 μg/mL and both 100 and 250 μg/mL concentrations.

Concentration μg/mL	Encapsulation efficiency percent Mean ± SD
100 μg/mL	72.3% ± 2.1%
150 μg/mL	81.5% ± 1.8%
200 μg/mL	77.8% ± 2.6%
250 μg/mL	73.4% ± 2.9%

These results confirm that the GE-NE system can effectively encapsulate cisplatin, with optimal loading observed at 150 μg/mL, providing a promising platform for further evaluation of its therapeutic and toxicological properties.

### 3.3 Physicochemical characterization of cisp-ge-ne

#### 3.3.1 Dynamic light scattering (DLS) analysis

The physicochemical characteristics of GE-NE and Cisp-GE-NE were evaluated using dynamic light scattering (DLS) at 37 °C. The initial mean particle size of the blank GE-NE was found to be approximately 120 nm, while the 135 nm for Cisp-GE-NE showed a slight increase, indicating successful drug loading and minor structural expansion of the droplets due to the presence of cisplatin. Particle size distribution (PSD) profiles also supported the monodispersity of the emulsions (Firoozi et al., 2020). To evaluate storage stability and droplet size, we further monitored both GE-NE and Cisp-GE-NE over 4 weeks under four different storage conditions: room temperature (light and dark) and 37 °C (light and dark). A gradual increase in droplet size was observed across all conditions, more pronounced in Cisp-GE-NE compared to blank GE-NE. The most significant instability was noted in the samples stored at 37 °C under light exposure, where the mean particle size of Cisp-GE-NE increased to approximately 165 nm by week 4, indicating light and heat-induced coalescence or degradation, as shown in Figure 1b. Conversely, emulsions stored at room temperature in the dark showed the highest stability, with only a marginal increase in size to 130 nm for GE-NE and 145 nm for Cisp-GE-NE.

Further, we also analyzed the zeta potential value of both GE-NE and Cisp-GE-NE. Our results were recorded as −28.5 mV, suggesting good electrostatic stability, while Cisp-GE-NEs exhibited a slightly reduced value of −25.2 mV, as shown in Table 3, still within the range indicative of stable colloidal systems. The PDI for both formulations remained below 0.3, indicating a narrow and uniform size distribution. Thus, our results suggest that drug loading slightly reduces nanoemulsion stability and that storage under cooler, dark conditions significantly preserves droplet integrity, which is crucial for maintaining therapeutic consistency in nanoemulsion-based drug delivery systems (Anarjan et al., 2014).

**TABLE 3:** Percentage retention of the active compound in the nanoformulation over 4 weeks under various storage conditions. The formulation was stored at room temperature (dark and light) and 37 °C (dark and light) and assessed weekly for stability, with Week 0 set as the baseline (100%). The nanoformulation demonstrated high stability under dark conditions at both room temperature and 37 °C, retaining over 93% and 86% of the compound, respectively, by Week 4. Notably, the formulation stored at 37 °C in the dark maintained 100% retention through Week 3, indicating strong thermal stability in the absence of light. In contrast, significant degradation was observed under light-exposed conditions, especially at elevated temperatures (37 °C), where the formulation began with only 86% retention at Week 0 and declined to 81% by Week 4. Similarly, light exposure at room temperature led to a gradual decrease to 85% over the same period. These findings underscore the sensitivity of the formulation to light and suggest that storage in dark, temperature controlled environments enhances its shelf life and integrity.

Storage conditions	Week 0	Week 1	Week 2	Week 3	Week 4
Room temp (dark)	100%	98%	96%	95%	93%
Room temp (light	100%	95%	91%	88%	85%
37 °C (dark)	100%	100%	100%	100%	86%
37 °C (light)	100%	92%	86%	83%	81%

#### 3.3.2 Fourier-transform infrared spectroscopy (FTIR) analysis

**FIGURE 2:**
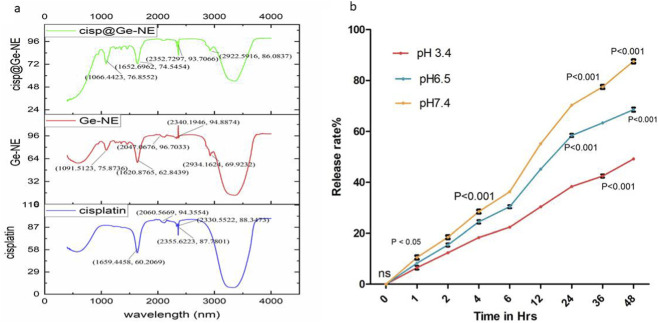
**(a)** FTIR spectral analysis of cisplatin, GE-NE, and Cisp-GE-NE. The FTIR spectrum of pure cisplatin shows characteristic peaks at 1,066, 1,455, 2060, 2,355, and 3,522 cm^-1^, corresponding to amine, NH bending, and Pt–N bond vibrations. GE-NE displayed peaks at 820, 1,091, 1,234, 1,628, and 2,941 cm^-1^, attributed to C–H stretching, OH bending, and ester/alkene groups. In the Cisp-GE-NE spectrum, the presence of both sets of peaks with slight shifts and reduced intensities confirms successful drug encapsulation and interaction, likely through hydrogen bonding or van der Waals forces. These spectral overlaps indicate the chemical compatibility and stable integration of cisplatin into the nanoemulsion system. **(b)** *In vitro* release kinetics of cisplatin from Cisp-GE-NE over 48 h at different pH levels (3.4, 6.5, and 7.4), measured as cumulative release percentage (%). The release rate significantly increased with higher pH: at 48 h, the release was 95.4% ± 2.1% at pH 7.4, 78.2% ± 1.9% at pH 6.5, and 52.6% ± 2.3% at pH 3.4. Statistical analysis using two-way ANOVA followed by the Bonferroni post hoc test revealed highly significant differences between the groups: P < 0.05 at 1 h, and P < 0.001 at all subsequent time points (2, 6, 12, 24, 36, and 48 h). The highest release at pH 7.4 suggests favorable drug diffusion in physiological pH, while reduced release at acidic pH implies pH-responsive control suitable for targeting the tumor microenvironment.

FTIR spectroscopy was performed to confirm the successful encapsulation and interaction of cisplatin within the Cisp-GE-NE, as shown in Figure 2a. The FTIR spectrum of pure cisplatin displayed characteristic peaks at 3,300–3,400 cm^-1^ corresponding to N–H stretching vibrations (Ali et al., 2026), 1,600–1,650 cm^-1^ attributed to N–H bending and Pt–N stretching, 600–800 cm^-1^ region ref, indicating the presence of Pt–Cl bonds. In contrast, the blank GE-NE showed key peaks at 2,920 and 2,850 cm^-1^ for aliphatic –CH_2_ and –CH_3_ stretching (Farshbaf-Sadigh et al., 2021), 1730 cm^-1^ for ester carbonyl (C=O) stretch from ginger oil components ref, 1,050–1,250 cm^-1^ corresponding to C–O–C and C–OH vibrations, A broad-band near 3,400 cm^-1^, attributed to hydroxyl groups from water or Tween 80. Upon encapsulation of cisplatin into the nanoemulsion, the FTIR spectrum of Cisp-GE-NE showed peaks at a noticeable shift or broadening of the N–H and Pt–N peaks, indicating possible hydrogen bonding or electrostatic interactions between cisplatin and surfactant/oil components (Ali et al., 2026). The reduction in intensity of the Pt–Cl bond region (600–800 cm^-1^) suggests interaction with the emulsion matrix. Retention of GE-NE peaks, especially around 2,920 and 1730 cm^-1^ (Farshbaf-Sadigh et al., 2021), confirms that the nanoemulsion matrix remained structurally intact. These results in a change of spectrum confirm the successful functionalization and encapsulation of cisplatin within the GE-NE matrix, likely involving non-covalent interactions that support drug loading without significant structural disruption of the emulsion.

#### 3.3.3 *In-Vitro* drug release kinetics

The release kinetics of cisplatin from the ginger oil nanoemulsion (Cisp-GE-NE) were evaluated in simulated gastrointestinal fluids at pH 3.4, 6.5, and 7.4 over 48 h using a dialysis method, as in Figure 2b. UV-visible spectrophotometric analysis of collected samples at (250–270 nm) revealed a sustained and pH-dependent release of cisplatin from the nanoemulsion matrix. At acidic pH, i.e., 3.4, the nanoemulsion system formulation showed a slower initial release, reaching approximately 45% cumulative drug release at 48 h. In contrast, at pH 6.5 and 7.4, a more enhanced and sustained release was observed, with 67% and 82% release, respectively. This suggests that the nanoemulsion system is stable in acidic conditions but efficiently releases the drug in near-neutral environments, mimicking physiological conditions.

#### 3.3.4 Stability assessment

The physical and chemical stability of the Cisp-GE-NE post-release rate was assessed over a 4-week storage period at room temperature and 37 °C, under both light and dark conditions. Particle size, color, phase separation, and residual drug content were monitored weekly. After 4 weeks, our results showed that there was no visible phase separation, and significant precipitation was not observed under any condition, confirming the physical stability of Cisp-GE-NE.At room temperature in the dark, over 93% of cisplatin remained encapsulated. At 37 °C in light, a moderate decrease in drug content was noted, with ∼81% drug retention, suggesting minor photothermal degradation. Detailed encapsulation efficiency is shown in Table 4. These findings confirm that the Cisp-GE-NE formulation is both stable and effective in releasing the drug in a controlled, pH-responsive manner, making it a promising candidate for targeted chemotherapeutic delivery. The detailed encapsulation efficiency (%) of Cisp-GE-NE over 4 weeks under different storage conditions is shown in Table 3.

**TABLE 4:** Morphometric analysis of Casper zebrafish larvae at 6 dpf following GE-NE-Cisp exposure.

Morphometric parameter	Control Mean ± SD	100 μg/ml Mean ± SD	%Change in comparison to control	p-Value	150 μg/ml Mean ± SD µg/ml	%Change in comparison to control	p-Value
Eye length	100 ± 3.4 µm	89.56 ± 3.6 µm	10.86% decrease	0.03	86.5 ± 3.8 µm	13.5% decrease	<0.001
Head length	180 ± 2.5 µm	-	-	ns	132.6 ± 3.7 µm	12.0% decrease	<0.01
Head width	86 ± 6.3 µm	75.63 ± 0.2.7 µm	10.6% decrease	>0.05	79.32 ± 4.2 µm	0.9% decrease	>0.05
Standard length	420 ± 4.2 µm	-	-	ns	358 ± 6.4 µm	12.45% decrease	<0.01

Morphometric parameters, including eye length, head length, head width, and standard body length, were measured in control and Cisp-GE-NE, treated groups at 100 μg/mL and 150 μg/mL. Values are presented as mean ± standard deviation (SD). Percent change is calculated relative to the control group. Statistical significance was assessed using One-way ANOVA., Significant reductions were observed in eye length and head standard length at the 150 μg/mL dose, suggesting concentration-dependent effects on craniofacial and axial development. The p-values indicate the level of significance (p < 0.05, p < 0.01, p < 0.001). No significant differences were found in head width.

### 3.4 Exposure and survival of zebrafish embryos to cisp-GE-NE

The Casper zebrafish embryos which are exposed to different concentrations of Cisp-GE-NE (100, 150, 200, and 250 μg/mL) exhibited a dose dependent effect on survival rates when exposed from 1 hpf to 6 dpf. From our results we have noticed that the Survival rates in the control group had remained high at 100% ± 2.1% throughout the exposure period, confirming the experimental conditions are optimal. Whereas in the treatment groups, survival rates have progressively decreased with increasing concentrations of Cisp-GE-NE. Embryos treated with 100 μg/mL and 150 μg/mL exhibited moderate reductions in survival, 92.7% ± 3.5% and 87.2% ± 4.9%, respectively, though these decreases were not statistically significant compared to the control group. However, a substantial decline in survival was observed at higher concentrations. At 200 μg/mL, the survival rate dropped to 78.5% ± 5.8%, and at 250 μg/mL, it further decreased to 56.3% ± 7.2%.

**FIGURE 3:**
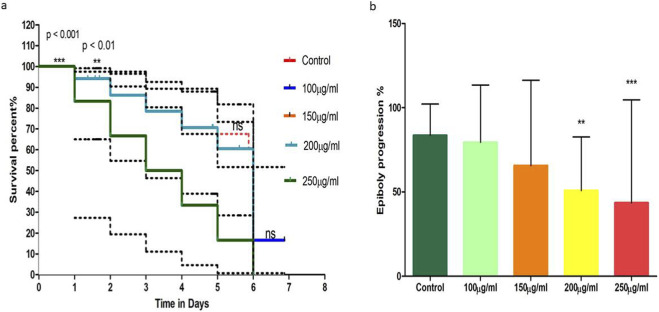
Impact of Cisp-Ge-Ne nanoemulsion system on zebrafish embryo survival and epiboly progression. **(a)** Kaplan–Meier survival curves of Casper zebrafish embryos exposed to increasing concentrations of Cisp-GE-NE (100, 150, 200, and 250 μg/mL) from 1 hpf to 6 dpf. The control group exhibited consistently high survival throughout the exposure period (100% ± 2.1%), confirming optimal experimental conditions. While embryos treated with 100 and 150 μg/mL showed only minor, non-significant reductions in survival (92.7% ± 3.5% and 87.2% ± 4.9%, respectively), significant dose-dependent declines were observed at higher concentrations. Survival dropped to 78.5% ± 5.8% at 200 μg/mL (p < 0.01) and further to 56.3% ± 7.2% at 250 μg/mL (p < 0.001), indicating a concentration-dependent toxicity of Cisp-GE-NE at doses ≥200 μg/mL. Survival analysis was performed using Kaplan-Meier curves and compared using the log-rank test. **(b)** Quantification of epiboly progression (%) at 8 hpf following 6-h exposure to Cisp-GE-NE. Embryos in the control group demonstrated normal epiboly movement (82.8% ± 4.6%), while those exposed to 100 and 150 μg/mL displayed slight, non-significant reductions (79.2% ± 5.3% and 67.8% ± 6.0%, respectively; p > 0.05). In contrast, embryos treated with 200 and 250 μg/mL exhibited significantly impaired epiboly progression (57.5% ± 7.6% and 48.5% ± 9.2%, respectively), with statistical significance indicated as p < 0.01 and p < 0.001. Data represent mean ± SEM from biological triplicates. Statistical analysis was performed using Kaplan-Meier curves and compared using the log-rank test.

Kaplan-Meier survival analysis followed by the log-rank test revealed that these reductions in survival were statistically significant at higher doses. Specifically, the 200 μg/mL group showed a significant reduction in survival compared to the control (p < 0.01), while the 250 μg/mL group exhibited a highly significant reduction (p < 0.001). These findings indicate that the Cisp-GE-NE nanoemulsion exerts a concentration-dependent toxic effect on zebrafish embryo viability, particularly at doses ≥200 μg/mL, as shown in Figure 3a.

### 3.5 Epiboly progression is inhibited by higher concentrations of GE-NE-cisp

Epiboly, an early and critical morphogenetic movement during zebrafish gastrulation, was analyzed at 8 hpf, in correspondence to 6 h of exposure to GE-NE-Cisp. A dose-dependent inhibition of epiboly progression was observed in the treated groups when compared to the control. Our results showed that the control group embryos exhibited healthy epiboly movement, with an average progression of 83.62% ± 2.86%, indicating normal developmental kinetics under the given experimental conditions. Embryos exposed to lower concentrations of Cisp-GE-NE, i.e., at 100 μg/mL and 150 μg/mL, had displayed slight and non-significant reductions in epiboly progression, with their mean values of 79.395 ± 5.263 and 65.705 ± 7.811, respectively, with a statistical p-Value of (p > 0.05), suggesting that these doses do not substantially interfere with early cell migration processes. In contrast, the embryos treated with higher concentrations demonstrated significant impairment of epiboly. At 200 μg/mL, the epiboly progression was reduced to 51.105 ± 4.4 (p < 0.01), and at 250 μg/mL, 42.555 ± 11.4, the progression further declined to 43.14% (p < 0.001) when compared to the control group. These statistically significant reductions highlight a clear inhibitory effect of Cisp-GE-NE on gastrulation at elevated concentrations.

The observed delay in epiboly indicates a disruption in coordinated cell movement, which is critical for proper embryonic patterning and development. These results underscore the developmental toxicity of Cisp-GE-NE concentrations less than or equal to 200 μg/mL (≥200 μg/mL), which has been considered for the post-experimental process as shown in Figure 3b. Statistical significance was determined using one-way ANOVA followed by Tukey’s post hoc test.

### 3.6 Teratogenic effects and morphological abnormalities

**FIGURE 4:**
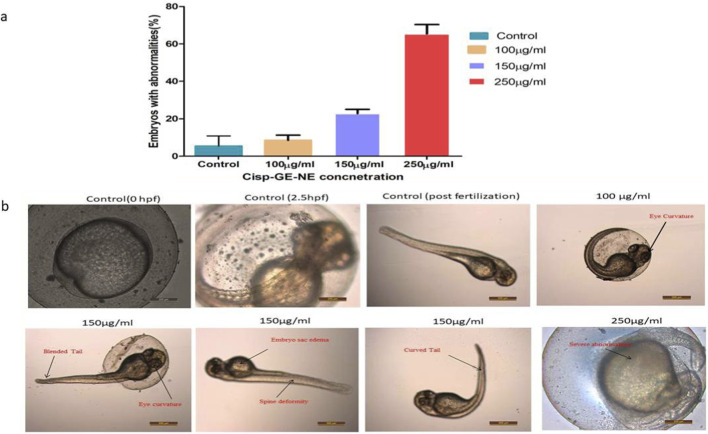
Teratogenic effects of Cisp-GE-NE in zebrafish embryos **(a)** A bar graph showing a clear dose-dependent increase in embryonic abnormalities after exposure to Cisp-GE-NE. At 250 μg/mL, 65% of embryos showed deformities, compared to 5% in controls. Lower concentrations (100 and 150 μg/mL) caused milder effects. **(b)** Representative images of zebrafish embryos across treatment groups. Control embryos showed normal development, while treated groups exhibited abnormalities such as eye curvature, spinal deformity, tail malformation, and yolk sac edema. The severity of these defects increased with concentration, with the most prominent changes at 250 μg/mL.

To observe the teratogenic activity of Cisp-GE-NE in the Casper zebrafish model, the zebrafish embryos were exposed to graded concentrations of Cisp-GE-NE (100, 150, and 250 μg/mL) daily to determine the dose-response relationship. Our results revealed that the teratogenic effects of Cisp-GE-NE increased in a concentration-dependent manner. Further, we also observed that the developmental abnormalities of adults began to appear as early as 48 hpf, and by 5 dpf, their frequency and severity had also increased. Across treatment groups, a range of morphological abnormalities was noted. These included spinal curvature (from mild axial bending to severe scoliosis-like distortions), pericardial edema, yolk sac edema, ocular defects (like microphthalmia or asymmetric eye development), and tail malformations (like kinking and truncation). These characteristics point to impaired organogenesis and disturbed embryonic development. By 5 dpf, we noticed that at least 65%of embryos had one morphological abnormality at the highest tested concentration (250 μg/mL), which was significantly higher than the 5% incidence seen in the control group (p < 0.001). Less noticeable effects were also seen at lower concentrations. Only 8% of embryos were impacted at 100 μg/mL, whereas 22% of embryos displayed obvious abnormalities at 150 μg/mL. According to these data, teratogenic effects become more common and severe above 150 μg/mL, indicating a threshold-dependent toxicological response. Figure 4a shows a quantitative analysis of the incidence of malformations across treatment groups, and Figure 4b shows representative photomicrographs of affected embryos at a 250 μg/mL concentration.

### 3.7 Hatching rates and morphometric analysis

**FIGURE 5:**
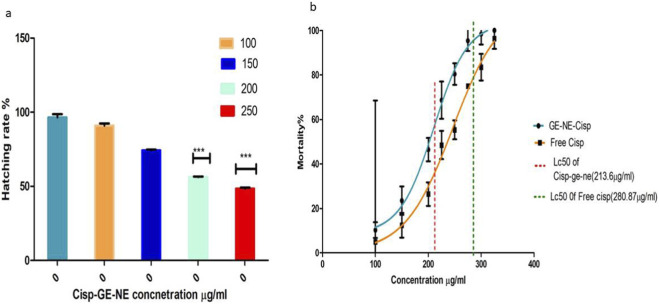
**(a)** Hatching rate of zebrafish embryos at 3 dpf following exposure to different concentrations of Cisp-GE-NE (100, 150, 200, and 250 μg/mL). A dose-dependent decline in hatching was observed, with significant reductions noted at concentrations ≥200 μg/mL. Data are presented as mean ± SD (n = 3 replicates). Statistical significance was analyzed, and values were compared to the control: ***p < 0.001. **(b)** Dose-response mortality curves for zebrafish larvae at 6 dpf treated with Cisp-GE-NE and free cisplatin. The LC_50_ of Cisp-GE-NE was 213.6 μg/mL (red dashed line), while that of free cisplatin was 280.87 μg/mL (green dashed line), indicating enhanced toxicity of the nanoemulsion formulation. Data are shown as mean ± SD.

Zebrafish embryos exposed to varying concentrations of GE-NE-Cisp were evaluated for hatching rates up to 3 dpf. Particularly at higher concentrations, a pronounced dose-dependent delay in hatching was noted. By 3 dpf, 95% ± 3.8% of the embryos in the control group had successfully hatched, demonstrating normal developmental progression. Likewise, there was no statistically significant decrease in hatching in embryos exposed to 100 μg/mL Cisp-GE-NE (90% ± 4.2%), whose p-value is >0.05, indicating that low concentrations had a milder effect on embryonic emergence. However, a more noticeable inhibition of hatching was observed as the concentration increased. When compared to controls, the hatching rate decreased to 73% ± 5.8% at 150 μg/mL, but this decrease was still statistically nonsignificant (p > 0.05). At 200 μg/mL, a notable reduction in hatching was initially noted, with only 52% ± 6.8% of embryos hatching by 3 dpf (p < 0.01). At 250 μg/mL, hatching was significantly hampered, with only 43% ± 7.2% of embryos successfully emerging from the chorion (p < 0.001), further exacerbating this effect. These results imply that Cisp-GE-NE disrupts zebrafish embryos’ typical hatching process in a concentration-dependent way. Developmental delay, chorion hardening, or physiological stress brought on by exposure to nanoparticles may be the cause of the impairment. A graphic representation of hatching rates for each treatment group is shown in Figure 5a, emphasizing the statistically significant drop that was noted at ≥200 μg/mL. Statistical significance was determined using one-way ANOVA followed by Tukey’s post hoc test.

### 3.8 B morphometric analysis

At 6 dpf, morphometric measurements were performed on Casper zebrafish larvae to assess the effects of GE-NE-Cisp exposure on development. Larvae from the 100 μg/mL and 150 μg/mL exposure groups were used as the subject of the analysis because more deformities formed in where untreated larvae acted as controls. Eye length (EL), head length (HL), head width (HW), and standard body length (SL) were among the measured parameters. The treated groups showed notable morphological changes, especially at the higher concentration, i.e., 150 μg/mL. In both treatment groups, eye length was significantly decreased by 10.8% in the 100 μg/mL group with p-value = 0.03 and by a more noticeable 13.5% in the 150 μg/mL group with a p-value <0.001. Similarly, there were significant decreases in head length (HL) and standard length (SL), with a 12% decrease seen at 150 μg/mL with p-value <0.01, suggesting compromised axial and craniofacial development. However, there was no significant difference in head width (HW) between the control and treated groups (p > 0.05), indicating that GE-NE-Cisp exposure had a greater impact on axial and anterior-posterior growth than on lateral cranial development. These results lend belief to the idea that exposure to GE-NE-Cisp during embryogenesis alters zebrafish larvae growth trajectories in a concentration-dependent way, especially influencing features like the head, eyes, and total body length, as shown in Table 4. Statistical significance was assessed using one-way ANOVA followed by Tukey’s post hoc test.

### 3.9 Median lethal concentration (LC_50_)

The median lethal concentration (LC_50_) of Cisp-GE-NE in Casper zebrafish larvae was determined using Probit analysis at 6 dpf, as shown in Figure 5b. The analysis yielded an LC_50_ value of 213.6 μg/mL, with a 95% confidence interval (CI) ranging from 198.4 to 218.6 μg/mL. In contrast, the LC_50_ for free cisplatin was calculated to be 280.87 μg/mL. This notable difference indicates that Cisp-GE-NE exhibits greater toxicity at lower concentrations compared to cisplatin in its unencapsulated form. The lower LC50 value observed for Cisp-GE-NE compared to free cisplatin suggests that nanoencapsulation alters the developmental toxicity profile of cisplatin in zebrafish embryos. These results strongly support the conclusion that encapsulation of cisplatin within the GE-NE nanoemulsion system significantly enhances its biological activity, as evidenced by the lower LC_50_ value. Thus, Cisp-GE-NE demonstrates greater efficacy and toxicity in the zebrafish embryo model, achieving 50% mortality at substantially lower doses than free cisplatin.

## 4 Discussion

The present work provides new information on the developmental toxicity of Cisp-GE-NE in the Casper zebrafish model, showing notable concentration-dependent effects on morphometric parameters, survival, and embryogenesis. Our results complement and go beyond earlier studies on the toxicity of cisplatin and drug delivery methods based on nanoemulsions. The significant decrease in hatching rate and compromised epiboly progression at concentrations of ≥200 μg/mL of Cisp-GE-NE is a crucial result of our investigation. This considerable decrease in hatching rate and delayed epiboly progression is consistent with the findings of Qiang et al. (2020), who found uptake of silver nanoparticles in zebrafish embryos accompanied by growth retardation and molecular responses (Qiang et al., 2020). Our results also show that higher concentrations of Cisp-GE-NE significantly increase teratogenic abnormalities, such as ocular defects, yolk sac edema, and spinal curvature. These findings are consistent with those of Hu et al., 2011, who examined biodegradable chitosan nanoparticles using a zebrafish embryo and discovered that, in contrast to free drug exposure, organ-specific toxicity and malformations were exacerbated by nanoparticle-mediated drug delivery (Hu et al., 2011). Although nephrotoxicity and neurotoxicity are already linked to free cisplatin in mammalian systems, our research suggests that nanoformulation may intensify these effects in developing embryos, possibly due to altered biological interactions associated with nanoencapsulation.

Interestingly, our morphometric analysis showed statistically significant decreases in standard length, head length, and eye length at 150 μg/mL Cisp-GE-NE, suggesting a disruption in axial and craniofacial patterning which are similar to studies by Gu et al. (2021), who showed that titanium dioxide nanoparticles caused comparable size reductions in zebrafish larvae, linked to increased apoptosis and disrupted cell proliferation, corroborate these findings (Gu et al., 2021). Notably, our study found no significant change in head width, implying that Cisp-GE-NE may be less likely to disrupt lateral cranial development, a detail that has received little attention in other toxicological studies and is worth investigating further. The lower LC_50_ value of Cisp-GE-NE (213.6 μg/mL) compared to free cisplatin (280.87 μg/mL) indicates increased toxicity due to the nanoemulsion system. These findings lend support to the hypothesis that, while nanoformulations improve drug delivery, they can also worsen toxicology. Ginger essential oil is well known for its anti-inflammatory and antioxidant properties, outcomes if not carefully calibrated, and several studies, including Abd Ali and Ismail, (2012), have proposed its role in mitigating chemotherapy-induced side effects (Abd Ali and Ismail, 2012). However, in our study, co-delivering cisplatin and ginger oil in a nanoemulsion matrix did not appear to reduce toxicity. This contradiction implies that the carrier oil’s beneficial properties may be overshadowed by cisplatin potent cytotoxic effects, particularly when administered via a highly penetrative nano-sized vehicle. It is possible that the nanoemulsion system may have altered the interaction of cisplatin with embryonic tissues. exacerbating rather than mitigating the toxic effects.

Overall, our findings support the use of zebrafish as a high-throughput vertebrate model for detecting subtle developmental toxicities. Their ability to show both gross morphological and behavioral phenotypes in response to environmental toxins makes them ideal for nanoformulation testing [40]. Importantly, the results highlight the need for rigorous toxicity testing of nanoemulsions, particularly those containing cytotoxic agents, before translation into clinical or environmental contexts.

## 5 Conclusion

This study demonstrates that cisplatin-loaded ginger essential oil nanoemulsion (Cisp-GE-NE) causes concentration-dependent developmental toxicity in Casper zebrafish embryos and larvae. Although low concentrations appear biocompatible, higher doses (≥200 μg/mL) significantly disrupt key developmental processes such as hatching, epiboly progression, and axial growth. Morphometric and teratogenic analyses confirmed the negative effects, which included increased deformities and impaired body patterning at high concentrations. The lower LC_50_ of Cisp-GE-NE compared to free cisplatin suggests increased toxicity, suggests that nanoencapsulation modifies the developmental toxicity profile of cisplatin in zebrafish embryos via the nanoemulsion vehicle. While this may improve cancer treatment efficacy, it also emphasizes the importance of conducting thorough toxicological profiling before repurposing such delivery systems for biomedical applications.

Overall, our findings from the study carried out emphasize the importance of balanced design strategies in nanoemulsion-based drug formulations, which maximize therapeutic effects while minimizing developmental risks. Future research should concentrate on long-term developmental outcomes, biodistribution studies, and comparative efficacy in tumor-bearing zebrafish models in order to fully assess the translational potential of Cisp-GE-NE formulations.

## Data Availability

The original contributions presented in the study are included in the article/Supplementary Material, further inquiries can be directed to the corresponding author.
